# Platelet-Rich Fibrin with Minocycline versus Arestin in Non-surgical Periodontal Therapy: A Split-Mouth Randomised Controlled Clinical Trial

**DOI:** 10.3290/j.ohpd.c_2810

**Published:** 2026-09-08

**Authors:** Nathan E. Estrin, Paras Ahmad, Savannah Laurey, Nima Farshidfar, Richard J. Miron

**Affiliations:** a Nathan E. Estrin Adjunctive Professor, The University of Iowa College of Dentistry and Dental Clinics, Iowa City, Iowa, USA; Department of Research, Advanced PRF Education, Jupiter, FL, USA; Private Practice, Lakewood Ranch Dental, FL, USA. Conceptualisation, methodology, formal analysis, investigation, resources, writing (original draft preparation), writing (review and editing), supervision, project administration.; b Paras Ahmad Researcher, Department of Research, Advanced PRF Education, Jupiter, FL, USA. Methodology, formal analysis, investigation, resources, writing (original draft preparation), writing (review and editing), supervision.; c Savannah Laurey Dental Hygienest, Private Practice, Lakewood Ranch Dental, FL, USA. Methodology, resources, supervision.; d Nima Farshidfar Researcher, Department of Periodontology, University of Bern, Bern, Switzerland. Methodology, investigation, resources, supervision.; e Richard J. Miron Researcher, Department of Research, Advanced PRF Education, Jupiter, FL, USA; Private Practice, Lakewood Ranch Dental, FL, USA; Department of Periodontology, University of Bern, Bern, Switzerland. Conceptualisation, methodology, formal analysis, investigation, resources, writing (original draft preparation), writing (review and editing), supervision, project administration.

**Keywords:** bone regeneration, guided tissue regeneration, microspheres, minocycline, platelet-rich fibrin

## Abstract

**Purpose:**

Periodontitis is a chronic inflammatory disease and while non-surgical periodontal therapy (NSPT) remains the first choice of treatment, its capability to completely resolve inflammation may be limited. Arestin® (1 mg minocycline microsphere) is an established adjunct, whereas platelet-rich fibrin (PRF) has recently emerged as a promising local drug-delivery scaffold. This split-mouth randomised controlled clinical trial compared the clinical efficacy of adjunctive minocycline delivered via conventional Arestin® versus incorporation within a PRF scaffold for the treatment of 4–6 mm periodontal pockets after NSPT.

**Methods and Materials:**

Fifteen participants were recruited, of whom 13 completed the 6-month follow-up. Each patient contributed two interproximal sites randomly allocated to receive either adjunctive PRF + Minocycline or adjunctive Arestin® after NSPT. Periodontal parameters, such as probing depth (PD), clinical attachment level (CAL), gingival recession (GR), and bleeding on probing (BOP), were measured at baseline and 6 months.

**Results:**

Both adjunctive approaches yielded statistically significant intra-group enhancements. Mean PD reductions were 1.13 ± 0.15 mm in the PRF + Minocycline group and 1.00 ± 0.67 mm in the Arestin® group. CAL gains were 1.02 ± 0.66 mm and 1.08 ± 0.61, respectively. Most sites in both groups showed CAL gains of 1 – < 2 mm (61.5%), whereas 15.4% of sites attained a residual PD of 3 mm at 6 months. Inter-group comparisons revealed no statistically significant differences for PD (*p* = 0.609), GR (*p* = 0.096), or CAL (*p* = 0.789).

**Conclusion:**

These findings suggest that PRF + Minocycline yielded comparable short-term outcomes to Arestin® as adjunctive treatments during NSPT. Therefore, treatment selection may be based on clinician preference, patient-related factors, cost, availability, and handling characteristics. Larger RCTs with longer follow-up are necessary to confirm these findings.

Periodontitis is a multifactorial, biofilm-mediated inflammatory disease characterised by a dysbiotic subgingival microbiota and a destructive host immune-inflammatory response, eventually resulting in gradual loss of the alveolar bone and periodontal attachment apparatus.^1,27
^ Non-surgical periodontal therapy (NSPT) encompasses mainly sub- and supragingival mechanical debridement (MD) with the utilisation of hand and ultrasonic instruments, and remains the standard, first stage of periodontal therapy intended to disrupt biofilm and control inflammation.^6,23
^ Although its efficacy is well-established, NSPT is often not sufficient as a solo treatment modality to entirely eradicate pathogens present within deep periodontal pockets, soft tissue microenvironments, and furcation areas, hence advocating for the application of adjunctive treatment approaches to promote clinical outcomes.^6,16,23,32
^ While NSPT remains the cornerstone of periodontal therapy, adjunctive therapies such as hard and soft lasers, biologics such as hyaluronic acid, and blood concentrates, as well as antibiotics and probiotics, are being investigated to improve local wound healing, patient-perceived pain, and reduce the need for further surgical therapy.^12,14,15
^


Over the past two decades, several adjunctive methods have been studied to improve the outcomes of NSPT, especially in sites with anatomically difficult root surfaces, deep periodontal pockets, and persistent inflammation.^12^ These adjuncts comprise autologous blood concentrates, hyaluronic acid, enamel matrix derivatives, host-modulation agents, laser-assisted periodontal therapy, photodynamic therapy, probiotics, and antibiotics.^15^ The justification for these treatments is to enhance bacterial alleviation, regulate the host inflammatory response, facilitate wound healing, and promote clinical outcomes beyond those obtained with MD alone.^14^ Existing evidence shows modest supplementary advantages for selected adjuncts, especially in moderate-to-deep periodontal pockets; nevertheless, the consistency and magnitude of these enhancements remain variable.^12,15
^ Resultantly, there is growing interest in formulating biologically based adjuncts that can concurrently offer antimicrobial function and facilitate periodontal wound healing while mitigating systemic adverse impacts.

Of these adjunctive strategies, locally administered biologics and antibiotics have gathered growing interest for their capability to target residual pathogenic microorganisms and mediate wound repair at the disease activity site. Local antibiotics, including minocycline hydrochloride,^10^ and autologous platelet concentrates (APCs), such as platelet-rich fibrin (PRF),^30^ have been widely investigated as adjuncts to NSPT owing to their localised therapeutic effects and feasible safety profiles. While systemic antibiotics have exhibited efficacy in inhibiting periodontopathogens, their regular clinical application is limited by numerous challenges, such as the requirement for extended or repeated administration, antimicrobial resistance development, potential systemic side effects, and variable bioavailability of the drug in the gingival crevicular fluid (GCF).^12^ Resultantly, locally administered antibiotics have emerged as a preferred alternative, enabling greater drug levels at the diseased area while diminishing systemic exposure.

Arestin® (minocycline hydrochloride microspheres) is one of the most extensively used local drug-delivery platforms in periodontal treatment.^12^ Williams and colleagues^40^ showed significant enhancements in probing depth (PD) alleviation and clinical attachment level (CAL) enhancements when Arestin® was utilised as an adjunct with NSPT, with feasible results also demonstrated in smokers.^28^


PRF is a second-generation APC consisting of a dense fibrin matrix in which growth factors, cytokines, leukocytes, and platelets are trapped, permitting a prolonged secretion of biomolecules that enhance immune regulation, tissue repair, and vascularisation.^3,22
^ The first-generation platelet concentrates, platelet-rich plasma (PRP), required the utilisation of anticoagulants to prevent clot formation, causing a liquid preparation.^8,31
^ Nonetheless, the presence of anticoagulants compromises wound repair and sustained growth factor release, hence limiting its regenerative potential.^13^


To overcome these drawbacks, PRF was introduced as a second-generation platelet concentrate formulated without anticoagulants, providing a solid fibrin scaffold able to progressively secrete sustained growth factors,^17^ while also exhibiting antimicrobial properties.^21^ Owing to its solid formulation, many studies have now demonstrated that PRF alone significantly improves NSPT when placed within a pocket following SRP.^5,9,24,25,26,29,34,35,38
^ Recently, injectable PRF (i-PRF) was introduced through modifications in centrifugation methods, enabling PRF to remain in a liquid form temporarily prior to its polymerisation.^18,19
^ This novel formulation has broadened the therapeutic application of PRF by allowing it to be utilised as a drug-delivery system with the ability to integrate and entrap small biomolecules prior to clot formation, efficiently transforming PRF into a bioactive local drug-delivery platform.^20^ Typically, PRF resorbs within a standard 2–3 week period; therefore, small biomolecules/drugs incorporated within its fibrin scaffold are slowly released over time within their local micro-environment during that 2–3 week timespan (similar timeframe as Arestin®). Research has shown the successful integration of extracellular vesicles, vitamins, and antibiotics into PRF matrices improves regenerative potential and antimicrobial potency.^7,33,36
^


Despite increasing evidence supporting the individual advantages of PRF and locally administered minocycline preparation, no study to date has attempted to utilise PRF as a drug-delivery system for the treatment of periodontal disease using NSPT in a similar approach to the locally delivered use of Arestin. Hence, this split-mouth randomised controlled clinical trial (RCT) aimed to assess and compare, for the first time, the clinical use of delivering 1 mg of minocycline using standard Arestin® versus its combined approach with PRF as adjunct interventions to NSPT in patients with Stage II or Stage III, Grade A or B periodontitis.

## Methods and Materials

### Ethical Considerations and Study Design

This study adhered to the CONSORT guidelines.^11^ The study protocol was prospectively submitted, reviewed, and approved by the Sterling Institutional Review Board (IRB approval ID: 12625-NEstrin) and registered on ClinicalTrial.gov (NCT: 07437209). This study was designed as a split-mouth RCT and performed in a private clinical practice (Lakewood Ranch Dental, FL, USA). All study protocols were conducted following the ethical considerations of the Declaration of Helsinki (2013 revision).^2^ Before enrollment, all participants received comprehensive written and verbal information associated with the study aims, clinical protocols, potential risks, and expected advantages. Written informed consent was obtained from all participants before starting any study-associated protocols, and participants’ confidentiality was maintained throughout the trial in accordance with applicable regulatory and ethical standards.

### Study Participants and Eligibility Criteria

A total of 15 systematically healthy individuals were consecutively recruited between December 2024 and April 2025. At the initial screening visit, each prospective participant underwent a thorough radiographic and periodontal assessment, including a detailed medical and dental history, full-mouth periodontal charting, and a complete series of bitewing and peri-apical radiographs, to confirm eligibility and periodontal diagnosis.

Inclusion criteria comprised: (i) adults aged 18–65 years; (ii) diagnosis of Stage II or III periodontitis, Grade A or B progression, as per the 2017 World Workshop classification, with a minimum of six interproximal sites showing PD > 4 mm (excluding third molars^39^; and (iii) presence of adequate number of qualifying interproximal sites to allow allocation to both treatment groups within the split-mouth design.

Exclusion criteria included: (i) previous periodontal treatment within the past 6 months; (ii) local or systemic antibiotic treatment administered within the past 3 months; (iii) diagnosis of Stage I/or IV periodontitis; (iv) poor oral hygiene defined as a full-mouth plaque score > 30%; (v) history of radiation or immunosuppressive therapy; (vi) presence of neurologic disorders, major mechanical obstruction to mouth opening, or acute temporomandibular capsulitis; (vii) systemic conditions that may compromise bone metabolism or wound healing such as metabolic bone disease, uncontrolled diabetes; (viii) history of substance abuse such as drug or alcohol; or (ix) current pregnancy, nursing, or planning pregnancy during the study period.

### Randomisation, Study Groups, and Baseline Clinical Evaluation

Individual patients contributed sites to both study groups, hence minimising confounding effects associated with inter-individual variability. A split-mouth randomisation strategy was used in which eligible interproximal sites were randomly assigned to one of the two adjunctive therapy groups utilising a computer-generated randomisation sequence, and allocation concealment was maintained until the intervention time.

The following two treatment modalities were assessed: (i) scaling and root planning (SRP) with adjunctive placement of Arestin®; and (ii) SRP with adjunctive application of PRF combined with minocycline (PRF + minocycline). At baseline, all clinical parameters were measured by a single trained investigator, such as PD, CAL, and gingival recession (GR).

### Intervention Protocols

#### Scaling and root planning

After administering local anaesthesia, all patients underwent full-mouth SRP utilising ultrasonic instrumentation and hand curettes. Adjunctive laser therapy was applied using a Fotona system (Ljubljana, Slovenia) and was specifically utilised to facilitate the eradication of residual inflamed granulation tissue within periodontal pockets. Root surfaces were thoroughly debrided until smoothness was confirmed with a periodontal explorer.

#### Arestin® application

For sites allocated to the Arestin® group, one randomly chosen interproximal site per patient received subgingival administration of 1 mg of minocycline (Arestin®), delivered utilising a pre-loaded, single-use syringe according to the manufacturer’s instructions.

#### PRF preparation and application

PRF was prepared immediately before the application, following the manufacturer’s instructions (Bio-PRF, bf-1, FL, USA). Two 10 ml solid-PRF tubes (red) and two liquid-PRF tubes (blue) were collected by venipuncture utilising a 21 G butterfly needle and centrifuged concurrently for 8 min at 700 × g employing a horizontal centrifuge system (Bio-PRF, bf-1, FL, USA) with a rotor radius of 13 cm. After collecting 2 cc of the PRF layer, it was transferred to a sterile metal dish, and 0.2 ml of minocycline in liquid form (10 mg/0.5 ml) obtained from a local pharmacy (Apothicare, Lakewood Ranch, FL, USA) (Fig 1) was mixed into 2 cc of liquid PRF before polymerisation at room temperature for 10 to 20 min, creating a cohesive PRF clot. After clotting, two to three small fragments of the clotted PRF were incorporated into the designated sites subgingivally utilising a cord packer.

**Fig 1 Fig1:**
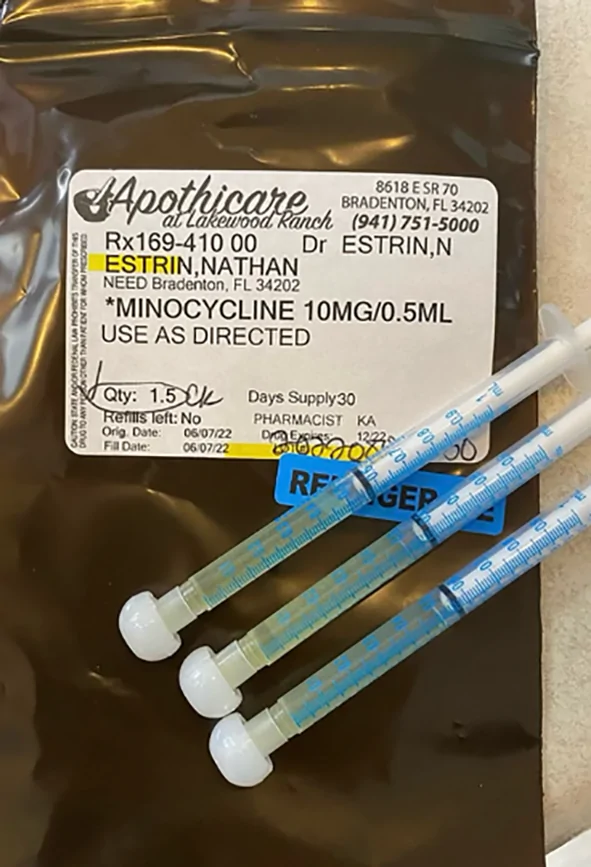
Example of a prescription from a local compounding pharmacy for the minocycline added to the injectable platelet-rich fibrin.

### Postoperative Care, Follow-Up and Outcome Assessment

After the completion of the assigned treatments, all patients received standardised postoperative instructions, such as reinforcement of oral hygiene measures and maintenance of routine plaque control practices. A follow-up visit at 2 weeks post-intervention was scheduled for professional reinforcement of oral hygiene instructions and assessment of soft tissue healing.

A supportive periodontal maintenance therapy was conducted at 3 months, comprising professional supragingival plaque removal and reinforcement of individualised oral hygiene measures. At the 6-month follow-up, all clinical periodontal parameters, such as PD, CAL, and GR, were re-evaluated and compared with baseline measurements to assess treatment outcomes.

### Power and Statistical Analysis

The sample size calculation was based on the primary outcome of CAL gain at the 6-month follow-up. The calculation used data reported by Sybil et al,^37^ in which the control group showed a standard deviation (SD) of 0.55. An online power analysis platform (Sealed Envelope Ldt., London, UK; https://www.sealedenvelope.com/power/continuous-superiority/) was utilised for the calculation. To account for methodological variations and provide a conservative estimate, an effect size (d) of 1.0 was selected. Based on a significance level of 5% (α = 0.05) and a statistical power of 80% (1-β = 0.80), the minimum required sample size was calculated as 13 participants. Permitting for an anticipated dropout rate of 20%, the final recruitment target was increased to 15 participants. Because of the split-mouth design, each participant contributed one site to each treatment group.

Statistical analyses were conducted using GraphPad Prism version 10.0 (GraphPad Software, San Diego, CA, USA). Continuous clinical variables (PD, CAL, and GR) were expressed as mean ± SD, while categorical variables (BOP) were expressed as frequencies and percentages. Data normality was assessed using the Shapiro–Wilk test. Given the split-mouth design, inter-group comparisons were conducted using paired statistical testing (paired t-test). All statistical tests were two-tailed, and a *p *value of < 0.05 was considered statistically significant.

## Results

### Primary Characteristics of Study Participants

Fifteen participants were recruited at baseline, consisting of six women and nine men, with a mean age of 46.7 ± 10.8 years (range: 30 to 63 years). Six patients (40%) were current smokers, while 9 (60%) had no history of smoking. As per the split-mouth study design, each participant contributed two qualifying interproximal sites, which were randomly assigned to receive adjunctive PRF combined with 1 mg minocycline or adjunctive Arestin® treatment. There were no adverse events or complications related to venipuncture, PRF preparation, or local material placement observed during the study period. The selected teeth were in both the maxillary and mandibular arches and involved premolars and molars, hence ensuring representation of anterior and posterior dentitions. During the study, one individual was lost to follow-up, and one additional individual was excluded due to inadequate plaque control, evidenced by a full-mouth plaque score of 100% at the 6-month maintenance visit. Finally, 13 patients were included in the final analysis.

### Periodontal Clinical Parameter Outcomes

#### Probing depth outcomes

Intra- and inter-group analyses demonstrated that both research groups yielded comparable clinical enhancements over the 6-month visit. PD significantly decreased within individual groups: from 5.08 ± 0.84 to 3.94 ± 0.69 mm in the PRF + Minocycline group (*p* < 0.001) (Fig 2a), and from 4.98 ± 0.90 to 3.98 ± 0.60 mm in the adjunct Arestin® group (*p* < 0.001) (Fig 2b), showing a mean difference of 1.13 ± 0.15 mm and 1.00 ± 0.67 mm, respectively. Inter-group comparison demonstrated no statistically significant difference in PD reduction at 6 months (*p* = 0.609; Fig 3a).

**Fig 2a to f Fig2atof:**
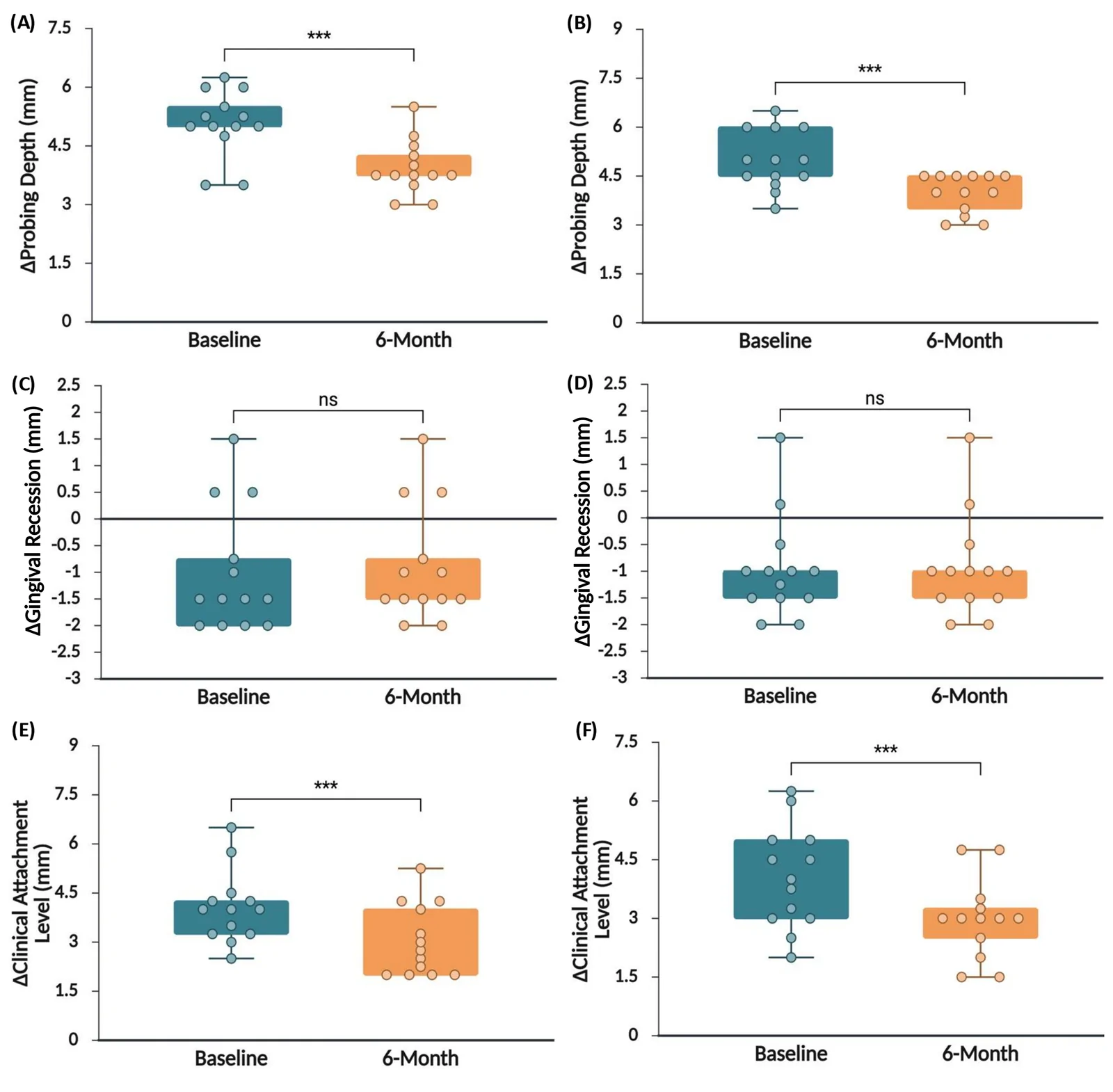
Probing depth values at baseline and 6-month follow-up for (a) PRF + Minocycline; and (b) adjunct Arestin® groups. Gingival recession values at baseline and 6-month follow-up for (c) PRF + Minocycline; and (d) adjunct Arestin® groups. Clinical attachment level values at baseline and 6-month follow-up for (e) PRF + Minocycline; and (f) adjunct Arestin® groups.

**Fig 3a to c Fig3atoc:**
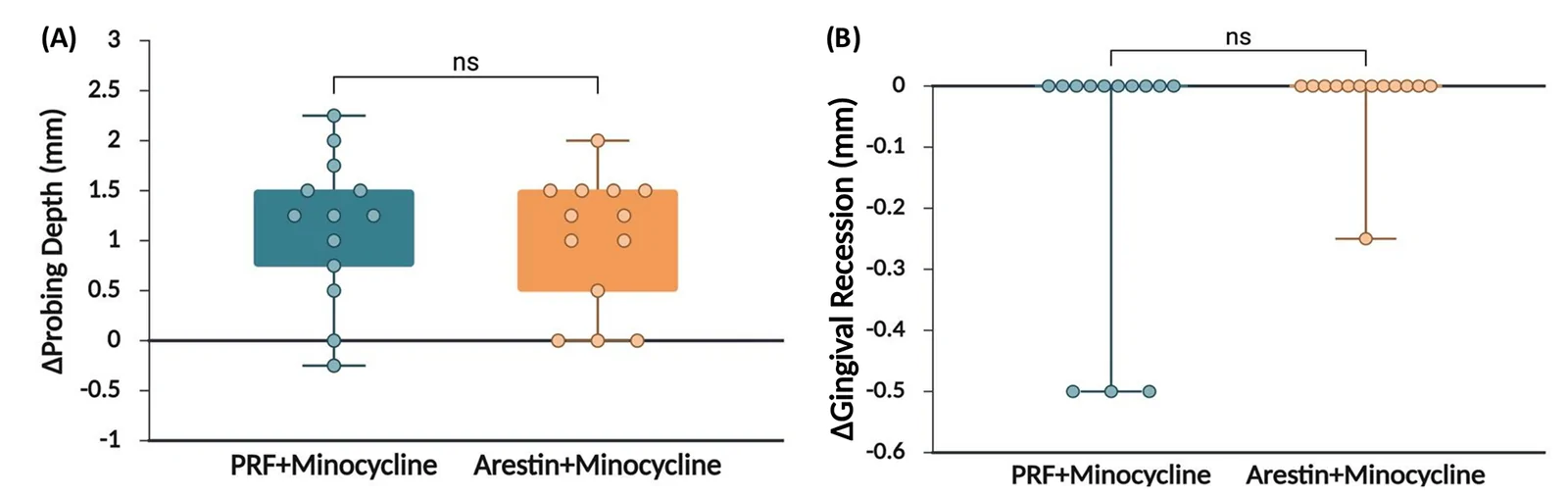

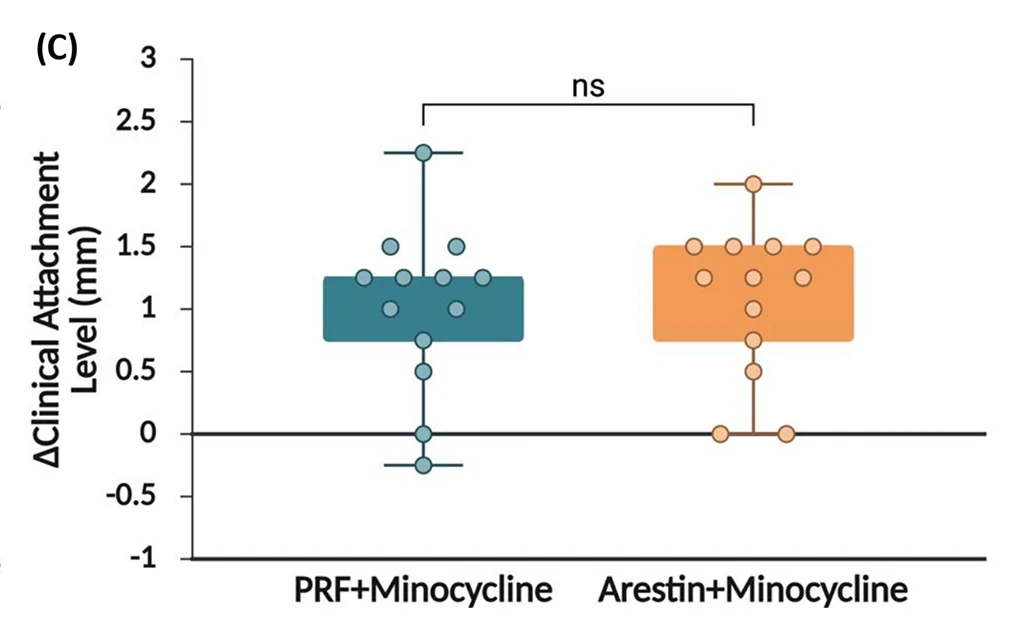
Mean changes in (a) probing depth; (b) gingival recession; and (c) clinical attachment level values between PRF + Minocycline and adjunct Arestin® groups.

Frequency distribution analysis of residual PD at the 6-month follow-up showed comparable outcomes between the two research groups. In the PRF + Minocycline group, two sites (15.4%) showed a residual PD of 3 mm, while 11 sites (84.6%) exhibited residual PD values > 3 mm. Similarly, in the Arestin® group, 2 sites (15.4%) showed a residual PD of 3 mm and 11 sites (84.6%) demonstrated residual PD values > 3 mm. No sites in either group showed residual PD values < 3 mm.

#### Gingival recession outcomes

GR mainly showed a stable trend across both research groups, with no statistically significant intra-group differences (PRF + Minocycline: *p *= 0.081 [Fig 2c]; Arestin®: *p* = 0.338 [Fig 2d]) and no significant inter-group differences at 6 months (*p* = 0.096; Fig 3b).

#### Clinical attachment level outcomes

Both groups exhibited significant CAL gains. CAL enhanced from 4.06 ± 1.09 to 3.04 ± 1.08 mm in the PRF + Minocycline group (*p* < 0.001) (Fig 2e) and from 4.06 ± 1.30 to 2.98 ± 1.01 mm in the Arestin® group (*p* < 0.001) (Fig 2f), with a mean difference of 1.02 ± 0.66 mm and 1.08 ± 0.61 mm, respectively. Inter-group comparison demonstrated no statistically significant difference at 6 months (*p* = 0.789; Fig 3c).

Frequency distribution analysis of CAL gain showed comparable clinical responses between the two research groups. In the PRF + Minocycline group, 4 sites (30.8%) gained < 1 mm of attachment, 8 sites (61.5%) gained 1 – < 2 mm, and 1 site (7.7%) gained 2 – < 3 mm, whereas no sites showed gains of ≥ 3 mm. A similar distribution was revealed in the Arestin® group, with 4 sites (30.8%) gaining < 1 mm, 8 sites (61.5%) gaining 1 – < 2 mm, and 1 site (7.7%) gaining 2 – < 3 mm, with no sites demonstrating gains of ≥ 3 mm.

#### Bleeding on probing outcomes

BOP was enhanced significantly in both study groups over the 6-month follow-up. In the PRF + Minocycline group, 100% of sites demonstrated BOP at baseline, which was alleviated to 30% at 6 months. Likewise, in the Arestin® group, all sites were BOP-positive at baseline, with a decrease to 18% of sites showing BOP at the 6-month follow-up (data not shown).

## Discussion

The present split-mouth RCT is the first study assessing the comparative clinical effectiveness of PRF in combination with minocycline versus commercially available minocycline microspheres (Arestin) as adjuncts to NSPT for patients with Stage II or III, Grade A or B periodontitis. The main outcomes showed that both adjunct therapies provided significant enhancements in PD, CAL, GR, and BOP over a 6-month follow-up, with statistically nonsignificant differences found between the therapeutic modalities. The findings suggest that PRF combined with minocycline produced short-term clinical outcomes comparable to Arestin® as an adjunct to NSPT for moderate periodontal pockets. These results directly address the existing gap in current published literature associated with PRF-based antibiotic delivery in comparison with well-established local antimicrobial platforms.

MD continues to be the standard of periodontal therapy; nonetheless, its intrinsic limitations in entirely eradicating pathogenic biofilms from complicated root anatomies and deep periodontal pockets have warranted investigation of adjunctive treatment methods. In line with the published literature,^41^ both treatment groups demonstrated a significant decrease in PD and enhancements in CAL, hence emphasising the well-established benefits of adjunct antimicrobial treatment when applied in combination with NSPT. The extent of CAL enhancement and PD alleviation observed in the Arestin® group is consistent with the outcomes reported by Williams and colleagues^40^ and other RCTs assessing minocycline microspheres, thus confirming the clinical effectiveness of the control treatment within the context of the present trial.

The PRF + minocycline group demonstrated comparable clinical enhancements, supporting the hypothesis that PRF can serve as an efficient bioactive drug-delivery platform. PRF provides a three-dimensional fibrin matrix capable of sustaining the secretion of leukocytes, cytokines, and growth factors, which may synergistically enhance immune regulation, wound repair, and tissue maturation after MD. The incorporation of minocycline into PRF before polymerisation helps localised antimicrobial administration while simultaneously leveraging the regenerative features of APCs, an amalgamation that may offer a biologically active carrier for local minocycline delivery; however, the present study cannot determine whether any additional effect was attributable to the PRF itself, the antibiotic, or their combination.

The significant reduction in BOP observed in both research groups further highlights the anti-inflammatory efficacy of adjunctive local interventions. Crucially, the PRF + minocycline group demonstrated a significant alleviation in BOP from baseline to 6 months, suggesting a substantial improvement in gingival inflammation over the 6-month follow-up. Since BOP is a well-established surrogate marker for disease activity and periodontal inflammation, these outcomes highlight the clinical importance of PRF-mediated adjunct treatment in diminishing the inflammatory burden.

Despite the promising biological rationale for PRF-based antibiotic administration, no statistically significant inter-group differences were found associated with PD decrease, CAL improvement, or changes in GR at 6-month follow-up. One plausible justification for this outcome is the incorporation of only moderately severe periodontal sites, with a mean baseline PD of around 5 mm. At this disease severity, NSPT alone is known to provide clinically meaningful and predictable enhancements, potentially restricting the ability to identify supplementary advantages conferred by adjunct modalities. This interpretation is supported by the classic research of Cobb et al,^4^ who showed that deeper periodontal pockets (> 7 mm) demonstrated significantly higher PD decrease after NSPT, while more modest enhancements were usually found in moderate defects. Hence, the limited potential for further pocket alleviation in moderately deep sites may have diminished the sensitivity of the current trial to identify subtle variations between the two adjunctive therapeutic strategies. Moreover, this may stem from the comparatively short follow-up duration, the limited sample size, and the high effectiveness of both treatments when applied together with adequate MD. Moreover, the split-mouth methodology, while advantageous for minimising inter-investigator heterogeneity, may reduce the capability to detect subtle differences between therapeutic strategies owing to potential crossover effects.

Clinically, the combination of PRF with minocycline may present several practical advantages, including potential economic benefits, the absence of synthetic carriers, its autologous nature, and the simultaneous administration of antimicrobial and regenerative signals. Moreover, PRF-based methodologies decrease issues related to foreign-body responses and may be particularly attractive to patients who prefer minimally invasive or biologically oriented therapeutic approaches. However, the supplementary procedural needs for blood collection and PRF formulation must be prudently considered against these potential benefits in a standard clinical setting. Utilising Arestin® offers a commercially standardised, ready-to-use local minocycline delivery system with minimal chairside preparation. Given the lack of significant differences between groups observed in this study, treatment selection may be based on clinician preference and considering factors such as time and clinical workflow, cost, material availability, and patient preference.

Numerous limitations of the present trial must be recognised. The relatively small sample size and single-centre framework may limit the applicability of the findings. Moreover, while the split-mouth design inherently controls for inter-individual variability and paired statistical analyses were used, the intra-subject association between treatment sites was not explicitly modelled utilising advanced statistical methods (generalised estimating equations or mixed-effects models). Hence, the likelihood of residual within-subject dependence cannot be completely ruled out and should be considered when interpreting the findings. Biochemical and microbiological evaluations were not performed, limiting the direct assessment of bacterial inhibition or regulation of inflammatory modulators. Another limitation was that adjunctive laser therapy was used in both groups as part of the NSPT protocol. Although this was standardised across treatment arms, it may have contributed to the observed clinical improvements and therefore represents a potential confounding factor when interpreting the isolated effect of PRF + minocycline or Arestin®. Furthermore, extended follow-up is necessary to validate whether the reported clinical improvements are sustainable over time and whether PRF-based delivery offers further clinical benefits/stability beyond a 6-month duration. Prospective RCTs with larger sample sizes, extended follow-up periods, and the incorporation of molecular and microbiological outcomes are recommended. Such investigations may offer deeper insights into the probable synergistic pathways of PRF-based antibiotic delivery and elucidate its function within evidence-based periodontal therapy.

## Conclusion

Within the limitations of the present study, it can be concluded that PRF in combination with minocycline demonstrated clinical outcomes comparable to Arestin® when used as an adjunctive to NSPT for the treatment of Stage II or III periodontitis. The PRF-mediated antibiotic administration platform represents a promising biologically oriented alternative that requires further investigation within periodontal therapy paradigms.

### Acknowledgements

#### Conflict of interest

Richard J. Miron holds intellectual property on the production of PRF and is the founder of Miron Research and Development in Dentistry LLC. All other authors declare that they have no conflict of interest.

#### Data availability statement

Data available on request from the authors.

#### Funding

This research was not supported by any funding.

## References
